# Multimodal intraoperative neuromonitoring in corrective surgery for adolescent idiopathic scoliosis: Evaluation of 354 consecutive cases

**DOI:** 10.4103/0019-5413.58608

**Published:** 2010

**Authors:** Vishal K Kundnani, Lisa Zhu, HH Tak, HK Wong

**Affiliations:** University Spine Center, National University Hospital, Singapore

**Keywords:** Neuromonitoring, scoliosis, somatosensory-evoked potentials, neurogenic motor-evoked potentials

## Abstract

**Background:**

Multimodal intraoperative neuromonitoring is recommended during corrective spinal surgery, and has been widely used in surgery for spinal deformity with successful outcomes. Despite successful outcomes of corrective surgery due to increased safety of the patients with the usage of spinal cord monitoring in many large spine centers, this modality has not yet achieved widespread popularity. We report the analysis of prospectively collected intraoperative neurophysiological monitoring data of 354 consecutive patients undergoing corrective surgery for adolescent idiopathic scoliosis (AIS) to establish the efficacy of multimodal neuromonitoring and to evaluate comparative sensitivity and specificity.

**Materials and Methods:**

The study group consisted of 354 (female = 309; male = 45) patients undergoing spinal deformity corrective surgery between 2004 and 2008. Patients were monitored using electrophysiological methods including somatosensory-evoked potentials and motor-evoked potentials simultaneously.

**Results:**

Mean age of patients was 13.6 years (±2.3 years). The operative procedures involved were instrumented fusion of the thoracic/lumbar/both curves, Baseline somatosensory-evoked potentials (SSEP) and neurogenic motor-evoked potentials (NMEP) were recorded successfully in all cases. Thirteen cases expressed significant alert to prompt reversal of intervention. All these 13 cases with significant alert had detectable NMEP alerts, whereas significant SSEP alert was detected in 8 cases. Two patients awoke with new neurological deficit (0.56%) and had significant intraoperative SSEP + NMEP alerts. There were no false positives with SSEP (high specificity) but 5 patients with false negatives with SSEP (38%) reduced its sensitivity. There was no false negative with NMEP but 2 of 13 cases were false positive with NMEP (15%). The specificity of SSEP (100%) is higher than NMEP (96%); however, the sensitivity of NMEP (100%) is far better than SSEP (51%). Due to these results, the overall sensitivity, specificity and positive predictive value of combined multimodality neuromonitoring in this adult deformity series was 100, 98.5 and 85%, respectively.

**Conclusion:**

Neurogenic motor-evoked potential (NMEP) monitoring appears to be superior to conventional SSEP monitoring for identifying evolving spinal cord injury. Used in conjunction, the sensitivity and specificity of combined neuromonitoring may reach up to 100%. Multimodality monitoring with SSEP + NMEP should be the standard of care.

## INTRODUCTION

Iatrogenic paraplegia resulting from surgical intervention of the spine is a devastating complication, and despite best practice in spinal deformity corrective surgeries, incidence range from 0.6 to 3.5%.^1^–^7^ Somatosensory-evoked potentials (SSEPs) are useful for monitoring dorsal column spinal cord function, and their use during correction of deformities has been shown to improve neurologic outcome.^8^–^11^ Although SSEP changes may reflect global spinal cord compromise in some clinical instances, impending damage limited to the motor tracts or anterior horn may go undetected. Somatosensory-evoked potential technique is specific only to the ascending dorsal tracts of the spinal cord and does not provide feedback on the integrity of the descending anterior motor tracts resulting in high false-negative rates^12^–^14^ resulting in heightened concern and debate about the adequacy of SSEP as sole modality of monitoring.^14^,^15^

Motor-evoked potentials (NMEP) can be reliably evoked by transcranial electrical stimulation of the motor cortex, which results in direct depolarization of the pyramidal tract neurons and conduction down spinal pathways. Evoked potentials are then recorded as a myogenic response in the form of a compound muscle action potential (CMAP) via needle electrodes placed in distal muscle groups of non-paralyzed patients.^14^–^16^ Neurogenic motor-evoked potential has been shown to have 100% sensitivity, however there are increasing reports of false-positive signal alerts.^17^

Neurogenic motor-evoked potential in conjunction with SSEP is recommended to improve the sensitivity and specificity of the neuromonitoring in scoliosis surgery. When used together, SSEP and NMEP permit sequential assessment of both the dorsal sensory and ventral motor columns, respectively.^16^,^18^–^20^ Continued advances in instrumentation and corrective techniques in the surgical treatment of scoliosis have further increased the need for such comprehensive monitoring.

The purpose of this study is to report the applicability, sensitivity and specificity of the monitoring methods in patients with a diagnosis of idiopathic scoliosis that underwent surgical correction at one institution. All patients in this study were monitored using SSEP and NMEP in combination. The intent is to evaluate the effectiveness of the protocol in the detection and prevention of neurologic injury for idiopathic scoliosis patients undergoing surgical correction.

## MATERIALS AND METHODS

We evaluated the prospectively collected neuromonitoring data of 354 consecutive operated cases of adolescent idiopathic scoliosis, from an ongoing prospective study, by independent observer (2004-2008).

Institutional review board approval was taken to undertake this study. Patients with established diagnosis of adolescent idiopathic scoliosis with age group (8 to <18 years) operated at single institution were included in this study. The patient of Secondary/non-idiopathic scoliosis, Age >18 years or < 8 years, Previous spine surgery, Associated Kyphosis and Abnormal preoperative neurological findings were excluded from the analysis

The analysis of prospectively collected medical records, intraoperative monitoring records, operative narratives, anesthesia records and outpatient clinical notes for all patients, who had undergone surgical correction of adolescent idiopathic scoliosis with multimodal monitoring (NMEP + SSEP), as per the laid protocol was undertaken. Important demographic and clinical data were documented including age, gender, height, weight and body mass index.^17^ Preoperative neurological status and preoperative curve type and degree were obtained from the outpatient clinical notes, and radiographic data was reviewed by independent observer. The operative reports, anesthesia records, spinal cord monitoring records were recorded prospectively and analysed to determine specific intraoperative events, loss in the amplitude of NMEP/SSEP, and the effect of interventions initiated to reverse those changes were noted. We followed the anesthesia and monitoring protocol, in conjunction with the published literature, on anesthesia and standard monitoring techniques.^16^,^21^,^22^ Multimodality spinal cord monitoring was achieved successfully in 354 consecutive patients, as a part of ongoing prospective trial, during surgical correction of adolescent idiopathic scoliosis, which formed the cohort of this study.

A uniform total intravenous anesthesia (TIVA) maintenance routine was implemented for all the patients so as to ensure minimal interference. Since the anesthesia maintenance protocol was the same for all the patients, induction with different drugs did not have any effect on final statistical outcome and monitoring alerts. Peripheral venous access often was accomplished with the assistance of nitrous oxide (60 to 70%) and a low-concentration potent agent (e.g., sevoflurane). Once peripheral venous access was established, anesthesia was induced either through potent mask anesthesia or with an intravenous agent. Mask induction was performed with the use of sevoflurane (6.0 to 8.0%) and nitrous oxide (60 to 70%) along with an opioid bolus (fentanyl, 2.0 to 3.0 mg/kg) and either a short-acting depolarizing (succinylcholine) or non-depolarizing (mivacurium) muscle relaxant. Following induction and intubation, all inhalational agents were turned off and no additional muscle relaxant was administered for the remainder of the surgery. Alternatively, intravenous induction was carried out with propofol (2.0 to 3.0 mg/kg) augmented with an opioid bolus and short-acting depolarizing or non-depolarizing neuromuscular blockade. An arterial line was placed along with stimulating and recording electrodes for neurophysiological monitoring following intubation. From this time forward, general anesthesia was maintained with pump-controlled intravenous infusions of propofol (125 to 200 *μ*g/kg/min) and remifentanil (0.1 to 0.5 *μ*g/kg/min) with particular effort made to achieve a stable, target mean arterial blood pressure of at least 65 mm Hg. A small (1.0 mg) dose of Versed was sometimes added as an adjunct for amnesia. No muscle relaxant was used following intubation so as not to compromise transcranial electric motor-evoked potential amplitudes.

### Monitoring

All spinal cord monitoring for this study was performed by one group of surgical neurophysiologists with a minimum of 4 years experience. Serial neurophysiological monitoring of spinal cord motor and sensory tract function was performed, from the time the patient was positioned, to the time patient was awakened from the anesthesia. Stimulus intensity was adjusted individually, ranging from 25 to 40 mA. Any further increments in current were not done. Repeated recording was done from both lower and upper-extremity efferent for transcranial electric motor potentials and afferent lower-extremity (posterior tibial nerve) and upper-extremity (ulnar nerve) somatosensory-evoked potentials. The upper-extremity transcranial electric motor and somatosensory-evoked potential modalities served both as neurophysiological controls to identify impending positional brachial plexopathy.^23^,^24^

#### Somatosensory-evoked potentials

Both cortical and subcortical somatosensory-evoked potentials were elicited by a 300-*μ*s square-wave electrical pulse presented, in turn, to the posterior tibial and ulnar nerves at a rate of 4.7/s. Stimulation intensity levels ranged from 25 to 45 mA, with intensity selected to achieve a response amplitude within the asymptotic portion of the somatosensory-evoked potential intensity versus amplitude curve for each individual patient. Cortical potentials were recorded from subdermal needle electrodes affixed to standard cranial locations and referenced as per international criteria^16^,^17^,^21^,^22^ of monitoring. All stimulation and recording of somatosensory-evoked potentials was performed with the use of commercially available neurophysiological monitoring workstations

#### Transcranial electric motor-evoked potentials

Transcranial electric motor-evoked potentials^16^,^17^,^21^,^22^ were recorded bilaterally from the first dorsal interosseous muscles in the upper extremities (control), and bilaterally from the anterior tibialis quadriceps and gastrocnemius muscles in the lower extremities. These myogenic responses were elicited with the use of a commercially available transcranial electrical stimulator that delivered a brief (50-*μ*s), high-voltage (250 to 500 V) anodal pulse train (two to seven pulses with a 1 to 5-ms interstimulus interval) between two electrodes (A-Gram, Glenn Rock, New Jersey) inserted subcutaneously over motor cortex regions C1–C2 (International 10–20 System). The stimulation parameter values (i.e., the number of pulses, interstimulus interval and voltage) were optimized to elicit maximal response amplitudes for each patient. Transcranial electric motor-evoked potentials were recorded with the same neurophysiology workstations used for somatosensory-evoked potential monitoring.

#### ‘Significant alert’

‘Significant Alert’, demanding reversal or intervention was defined as persistent (>1 occasion of NMEP stimulus or >10 min of SSEP change) loss of 65% of amplitude (unilateral or bilateral) of the transcranial electric motor-evoked potentials or ≥50% of the amplitude of the somatosensory-evoked potentials relative to a stable baseline. Increase in the latency by more than 10% was also considered significant alert to prompt intervention.^16^,^17^,^25^

#### Intervention

Any significant alert triggered a sequence of interventional steps in accordance with international consensus.^16^,^25^,^26^,^27^ If the neurophysiological change was in the time-frame of a specific surgical maneuver, the precipitating maneuver was promptly reversed. Regardless of whether the change was related to a particular surgical action, the anesthesiologist was always directed to raise the mean arterial blood pressure in order to promote better spinal cord perfusion. Temperature and oxygen saturation were double checked. The technician was prompted to ensure that the circuit is in line with no disconnection of wires to the amplifying terminal. After temporary cessation of the surgery and institution of hemodynamic management, if there was no reversal of signals, corrective forces were reversed and a methylprednisolone bolus of 30 mg/kg was administered, to restrict the effect of cord edema. If the amplitude still did not improve, even after reversal of correction and implant removal, cessation of the procedure was considered. If amplitude returns to normal with the above-mentioned interventions, arthrodesis in the safest position was accomplished.

### Statistical analysis

We defined an impending injury as any important neurophysiologic change that prompted some type of intervention. Any decline in >1 motor power grade was considered as new onset neurodeficit. All patients were divided into four groups:

- Significant alerts in SSEP
- Significant alerts in NMEP or
- Significant alerts in both SSEP and NMEP,
- Without neuromonitoring alerts.

Correlation of significant alerts to neurodeficit was done by dividing these four groups into a) postoperative neurodeficit or b) No neurodeficit postoperatively.

The accuracy of the monitoring with regard to detecting impending iatrogenic spinal cord injury was expressed by standard statistical measures utilized for diagnostic tests, including sensitivity, specificity and positive predictive value by standard statistical measures. For the purposes of this study, definitions related to sensitivity and specificity [Table 1] of the SSEP and NMEP were related and modified with operational definitions of new-onset injury as published by Hilibrand *et al*.

**Table 1:** Definitions of statistically significant alerts

True-positive alert	Significant alert in NMEP/SSEP signals indicative of an ‘evolving’ injury that (1) was irreversible despite all interventional measures and was followed by a postoperative neurologic deficit or (2) responded favorably to intervention (improved to within 25% of the initial stable baseline value)
False-positive alert	Significant alert that could not be reversed to within 25% of the stable value, but the patient awoke without any postoperative sensory and/or motor deficit
True-negative alert	No critical changes and the patient awoke neurologically intact
False-negative alert	Patient awoke with a new neurologic deficit with (1) No significant change in NMEP/SSEP (2) a relevant signal change had resolved to within 25% of baseline following intervention

## RESULTS

There were 309 female patients, 45 male patients ranging in age from eight to eighteen years (average, 13.6 years old) at the time of surgery. Preoperative Cobb's angles ranged from 40 to 138°. The clinical demographics, curve pattern, magnitude of the curve and surgical procedures undertaken are summarized in Tables 2 and 3.

**Table 2:** Demographic data of AIS cases undergoing neuromonitoring in relation to significant alerts

	Without signal alerts	With signal alerts
Age	13.6 years (8–18 years)	14.1 years
Sex	M : F = 42 : 299	M : F = 3 : 10
Average weight	41 kg	37 kg
Average height	131 cm	135 cm
Body mass index	23.5 (21–28)	23.9
Curve characteristics		
Average magnitude	48° (40–108)	55° (45–89)
Average Rissers	Grade 3	Grade 3
Curve type (Lenke)		
Type 1	112	2
Type 2	23	3
Type 3	78	3
Type 4	16	2
Type 5	67	1
Type 6	45	2

**Table 3:** Surgical procedures performed

Total	354
Anterior	30
Posterior	302
Anterior + posterior	22

Preoperative baseline monitoring with the standard neuromonitoring protocol were available in all the patients.Seven patients did not elicit neuromonitoring signals at lower currents (25 mA) and baseline signals were obtainable only with higher current values (30–40 mA).

A total of 341 out of 354 patients did not show any signal alert and had no postoperative deficit, and were classified as true negatives. There were five cases with non-significant alert, mostly asymmetrical changes in limbs during instrumentation, which progressively returned to normal values over 30 min without intervention. In this case, latency and amplitude of SSEP were affected less compared to NMEP. Because this change did not exceed the traditional 50% criterion and showed an immediate spontaneous trend toward improvement, there was uncertainty about its significance, and no surgical intervention apart from a temporary pause was undertaken. No new postoperative event, sensory or motor, was detected in these cases and was considered true negatives.

However, 13 patients (3.6%) (3 male, 10 female) with signal alert met the criteria of significant alert. There was no significant gender, height, weight or BMI predilection to develop signal alert. Out of 13, all had signal alerts in NMEP; 8 had change in both NMEP and SSEP.

Of the 13 patients with substantial NMEP or SSEP signal alert [Table 4], signal alerts were reversible in 9 patients, coming back completely within normal range and patients not having any neurological deficit postoperatively. Four patients did not show any reversal after systematic intervention. Two had neurological deficit, while two had no neurodeficit despite persistent abnormal signals. Cause–effect relation was established in 11 patients and was related to sudden drop in mean arterial pressure in 4 patients, surgical corrective maneuver in 6 patients and critical breach due to inadvertent medial insertion of screw in low thoracic vertebrae (T11) in one patient. No established cause–effect could be established in two cases (signal alert detected during preparing host bed for bone graft in one, and during final tightening maneuver in other). Among both these patients, SSEP signals were stable, and despite aggressive intraoperative corrective interventions no recovery of signals was noted. However, these patients did not have any neurological deficit after surgery and were classified as false positives.

**Table 4:** Significant neuromonitoring alerts

	Only SSEP alert	Only NMEP alert	SSEP + NMEP alert	No SSEP/NMEP alert
Without	0	5	6	341
postoperative				
neurological				
deficit				
With	0	0	2	0
postoperative				
neurological				
deficit				

SSEP - Somatosensory-evoked potentials, NMEP - Neurogenic motor-evoked potentials

Of 11 patients with significant alerts and definite cause–effect relationship, eight had significant NMEP + SSEP alerts during surgery. In all the eight patients in whom major changes were detected by both modalities, the SSEP changes lagged behind the NMEP changes by an average of 17 min. Hence, although the specificity of the SSEP monitoring was equivalent to that of the NMEP monitoring, the temporal differences were clinically important [Table 5].

**Table 5:** Sensitivity and specificity of neuromonitoring in AIS

	SSEP %	NMEP %	SSEP + NMEP %
Sensitivity	51	100	100
Specificity	100	96	99

**Figure 1:**
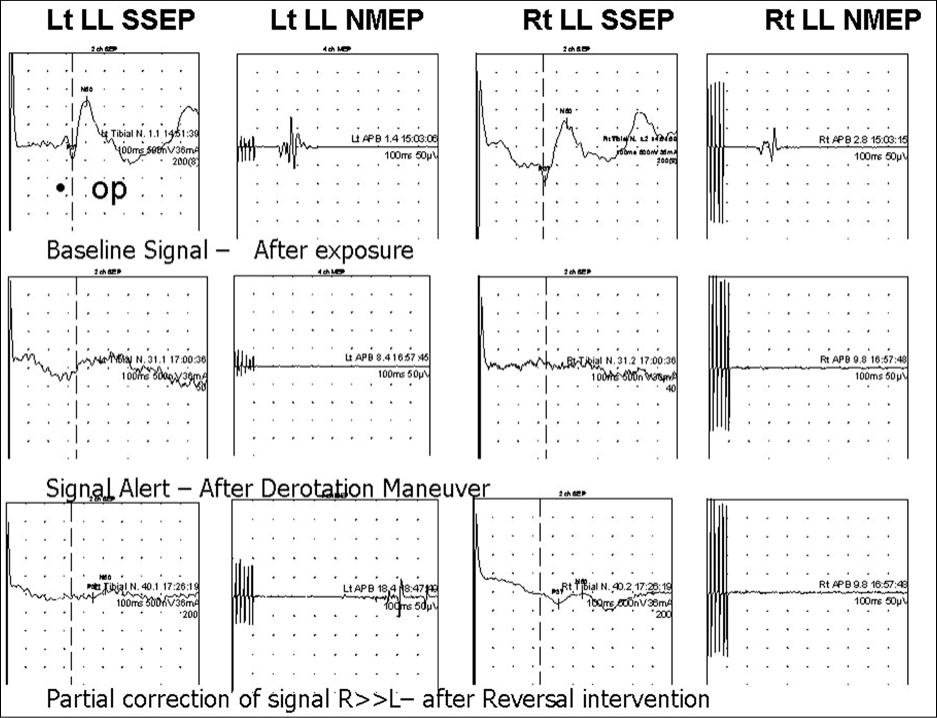
A case of a 16-year-old female patient with double major curve (Lenke type) right thoracic T3–T11 = 86° curve and thoracolumbar T11–L4 = 78° curve with normal baseline monitoring parameters. Significant alert was noticed with decline in both SSEP and NMEP signals during intraoperative corrective maneuver. Reversal action was started. However, only partial recovery of signals was detected. Patient had postoperative neurological deficit (Paraparesis - Frankel B)

Two of the thirteen with significant alert developed neurological deficit. In first case alert related to corrective maneuver on double major curve was followed by signal alert, and reversal was not met with complete recovery of signals [Figure 1] and arthrodesis was carried out with minimal correction. Patient awoke with paraparesis postoperatively (Frankel B), which improved subsequently to Frankel C at final follow-up. The other had transient, but clinically evident, lower-extremity weakness resolving over a period of 12 weeks. In the other case critical breach of screw was associated with signal alert and hypotension, and with removal of screw reversal of signals was observed, however only to 30% of baseline, patient had postoperative weakness of ipsilateral lower limb muscles resolving over a period of time. Both these patients with neurodeficit had NMEP + SSEP signal alerts and were classified as true positives.

Five patients with complete loss of NMEP signals, but stable SSEP amplitudes having definite cause–effect relationship and signals reversed with intraoperative intervention, were also classified as true positives. In two patients, NMEP amplitude changes responded to an increase of the mean arterial pressure to 90 mm Hg or more, and the administration of a methylprednisolone bolus. Two patients had temporary release of correction and subsequent attempts having no significant alerts, both underwent planned correction and had no postoperative deficit. One patient revealed reversal only after persistent reversal of correction and underwent the arthrodesis in safe position.

## DISCUSSION

In 1992, the Scoliosis Research Society issued a position statement^20^ regarding the use of neurophysiologic monitoring during spinal surgery. They concluded that, ‘A substantial body of research has demonstrated that neurophysiologic monitoring can assist in the early detection of complications, and can possibly prevent postoperative morbidity in patients undergoing operations on the spine. The Scoliosis Research Society considers neurophysiologic monitoring a viable alternative, as well as an adjunct, to the use of the wake-up test during spinal surgery.’

The use of a wake-up test alone has many well-documented limitations.^10^,^16^,^17^,^28^ It poses certain risks to the patient, such as inadvertent extubation, possible loss of intravenous lines, or recall. More important, it does not pinpoint the time or onset of neurologic injury. In this study ‘Wake-up test’ was not done, in accordance with the above-mentioned reasons. Also with no muscle relaxants used (due to known interference with NMEP signals), deep anesthesia was maintained and it could have been time-consuming and cumbersome to have frequent wake-up tests.

The goal of neurophysiologic monitoring is rapid detection of any neurological insult that can result in neurological deterioration during surgical intervention on the spine and prompt early intervention to systematic thus reversing the insult and avoiding adverse sequels.^16^,^29^ In the present study, there was no case that had no signal change in SSEP and NMEP both, and still developed neurological deficit (false-negative monitoring). Our study supports that multimodality neuromonitoring of spinal cord sensory and motor function, during surgical correction of adolescent spinal deformity is feasible and provides useful neurophysiologic data to reverse neurological insult.^30^,^31^ In view of high false-negative associated with SSEP^32^,^33^ isolated SSEP monitoring is not the standard of care anymore.^34^,^35^ With previous reports of high sensitivity of NMEP, combined multimodal monitoring makes this modality more reliable to avoid neurological sequels.^33^,^36^ Debate continues about the various methods for eliciting MEPs, electrical versus magnetic and spinal versus cortical stimulation; to name a few, in the present study, combined intraoperative neuromonitoring was utilized for all the cases. Regardless of method, the use of NMEP techniques is thought to provide additional information not obtained with SSEP concerning the integrity of all neurological tracts of the spinal cord.^18^,^33^,^36^

The combined use of SSEP and NMEP for intraoperative monitoring is thought to provide the most comprehensive information on the status of the spinal cord.^27^,^28^ Thirteen of 354 patients had significant alert with direct cause–effect relation in 11 cases (true positive). With prompt reversal intervention, the adverse sequel could be avoided in 11 cases. However, if SSEP alone was used then only half of them could not have been discovered intraoperatively resulting in higher neurological complication rates. Combined multimodal intraoperative monitoring during scoliosis surgery should be the standard of care.^28^

Although SSEP complement NMEP, the necessary averaging introduces a feedback delay. In the present study, patients with neurological deficit had significant and concomitant SSEP + NMEP amplitude loss, stating improved sensitivity of multimodal monitoring protocol. However, the SSEP change lagged behind the onset of the changes by 17 min and is attributed to the multiple signal stimulation protocol, which is established downside of SSEP monitoring. This phenomenon of lag in signals with SSEP has been supported by previous studies.^7^,^9^,^14^

Pelosi *et al.*,^37^ were able to achieve combined monitoring in 104 of 126 procedures (82%) in 97 patients (mean age: 21.7 years ± 13.9; 79 spinal deformity; 18 miscellaneous disorders). They found significant intraoperative electrophysiological changes in one or both methods in 16 patients – SSEPs recovered in 8 of 8 and NMEP in 10 of 15 (67%). There were new postoperative deficits in 6 of 16 with abnormal testing. They concluded that combined monitoring was safe, reliable and sensitive. Of the 354 patients in this study, two presented with a new-onset neurologic deficit postoperatively, for an incidence of 0.54%. The difference in neurologic sequelae can be explained due to mixed subset of patients, and in the present study, percentage of postoperative neurologic deficit is in keeping with previously reported studies using multimodal neuromonitoring for idiopathic scoliosis, but better than those where only SSEP or no neuromonitoring was employed. None of the patients in this study had isolated SSEP alert, but 5 patients had a detectable NMEP alert with stable SSEP values, further reinforcing the pitfalls of isolated SSEP monitoring. All the NMEP alerts were reversible with prompt intervention. None of the patients with isolated NMEP alert had new neurological deficit; this can be explained by higher sensitivity of this modality resulting in early detection of physiological insult (suspected vascular event) with increased safety during corrective procedure.

In a large review of 1445 anterior cervical surgical procedures, Lee *et al.*^38^ reported a false-positive rate for MEP alerts of 5.8% based. However, of 145 cases associated with a major alert, only two were associated with a new postoperative deficit. The authors assumed that the vast majority of alerts reflected an impending cord injury that was prevented by systematic intervention. In the current study, the false-positive rate was 11% and we agree with the hypothesis that aversion of impending spinal cord injury is attributed to systematic intervention. However, there have been contradictory reports as well.

Literature studies assess monitoring outcome through true positive, false positive, true negative and false negative.^17^,^18^,^37^,^39^ Nevertheless, they differ in their definition of what is a false positive and true positive.^16^,^17^ Cases in which a significant fall in evoked potentials is not followed by postoperative neurologic deficit are considered false positive by some authors, even if correlated with an intraoperative event considered at risk for spinal cord function. The study by Hilibrand *et al.*,^28^ included in definition of true positive “any case in which significant loss of potential was reversed by an intervention”. We agree with the philosophy that such cases may represent an alert due to temporary but consequential change in cord physiology that would not have resulted in an observable neurologic deficit had the patient been unmonitored, and so true positive. Also if the signal alert was false positive that should either resolve of its own due with no intervention or should be nonreversible with no subsequent neurological deficit, as in two cases in this study. Also, further analysis of the cause–effect correlation increases the reliability of the signal alerts, adding to the validity of definitions of true- versus false-positive alerts.

Franck *et al.* performed multimodality monitoring in 191 patients (90 - idiopathic, 79 - NM, 22 - miscellaneous) with a mean age of 15 years. They reported baseline SSEPs in 173 of 191 (90.06%) and baseline MEPs in 174 of 191 (91.1%). In our study, the baseline monitoring values were obtainable in 100% of cases. Franck *et al.* had 5 true positives, 6 (3.4%) false positives and no false negatives. Their overall sensitivity was 100%, and 52.69% specificity. In our group, there were 11 true positives and 2 (0.5%) false positives. Somatosensory-evoked potential although have the equipotent specificity (100%) for detecting neurologic compromise, but a low sensitivity (51%) in patients undergoing spinal deformity surgery.

Large discrepancies in the reported sensitivity and specificity of spinal cord monitoring^16^–^18^,^28^,^39^,^40^ among previous studies are largely due to different definitions of true and false-positive alerts. In our study, the sensitivity and specificity were calculated for both the modalities and for multimodal monitoring for comparative purposes. The sensitivity, specificity of SSEP and NMEP are 51%, 100% and 100%, 95%, respectively, which are in compliance with the published results on multimodal monitoring. However, the specificity is 99% and sensitivity is 100% of multimodal monitoring using NMEP + SSEP, which is better than the reported results in literature. This change is due to lesser numbers of false positives in present study, and further elucidates the efficacy of our protocol in monitoring. The positive predictive value of multimodal monitoring was significantly high for the same reasons in present study. Monitoring of electric motor evoked potentials was 100% sensitive in identifying patients who subsequently awoke with neurological deficit, whereas the overall sensitivity of SSEP remains 51%, although with high specificity (100%).This finding is entirely consistent with other studies.

Attempts were made to study the preoperative variables and risk factors associated with monitoring alerts. Due to small numbers of true positives, significant association of variables like Age, Sex, BMI and curve magnitude is challenging. Although significant association could not be associated with any of the preoperative variables, signal changes were associated with corrective maneuver in 6 of the 11 cases. Another significant finding was the role of hypotension to trigger the signal alert, which was seen in 5 of 11 cases. However, most of our surgeries were carried out under mean arterial mean pressure of 65–80, with no signal alerts, sudden/gradual drop in blood pressure may lead to signal alerts, and should be sought for incorrective surgeries. This poses a challenge to initiate further studies, to identify patients who are at risk of suboptimal perfusion with hypotensive anesthesia. Therefore special attention must be given ensuring adequate spinal cord perfusion in all patients.

There are limitations of this study. Firstly all cases with persistent significant alert were promptly tackled with reversal intervention with no observational period. In this attempt, the chances of spontaneous reversal were not studied, resulting in higher number of true positives. Pelosi *et al.*^37^ highlighted that the possible drawback of MEPs could be because of the higher number of perhaps unnecessary alarms. In their study, the authors suggested ‘transient’ changes of MEPs with normal SSEPs were probably of no clinical significance. In this study, intervention was done in only cases with ‘persistent’ change even when in any single modality, as any delay in intervention after neurological insult detected by monitoring was not ethical. Secondly, no attempt was done to evaluate the ‘delayed’ postoperative neurologic deficits as there is little evidence in consensus with philosophy of few authors that physiological or vascular insult due to stretching, during spinal instrumentation and or prolonged intraoperative hypotension, can lead to postoperative spinal cord swelling, compromising vascular supply. Although these concerns are genuine, but lack of evidence and lack of evidence based reports in contemporary literature cannot be neglected.

Further studies may assist understanding of cord perfusion and relation to physiologic insult with corrective maneuver.

## CONCLUSION

Results of this study show that (1) Neurogenic motor-evoked potential (NMEP) is highly sensitive, more than SSEP in detecting evolving spinal cord injury during corrective scoliosis surgery. (2) Somatosensory-evoked potential monitoring complements transcranial electric motor evoked potential monitoring by being highly specific to physiologic/mechanical insult and highly sensitive to posterior column. (3) Early detection through significant alert with neuromonitoring, offers an opportunity for rapid intervention to prevent injury progression and possibly reverse impending neurologic sequel. (4) Multimodal monitoring enhance safety in deformity surgery and should be the standard of care..
